# Analysis of inter-patient variations in tumour growth rate

**DOI:** 10.1186/1742-4682-11-21

**Published:** 2014-05-20

**Authors:** Esmaeil Mehrara, Eva Forssell-Aronsson

**Affiliations:** 1 Department of Radiation Physics, Institute of Clinical Sciences, Sahlgrenska Cancer Center, Sahlgrenska Academy, University of Gothenburg, Göteborg SE - 413 45, Sweden; 2 Department of Medical Physics and Biomedical Engineering, Sahlgrenska University Hospital, Göteborg, Sweden

**Keywords:** Tumour, Growth rate deceleration, Cancer, Modelling, Gompertzian

## Abstract

**Purpose:**

Inter-patient variations in tumour growth rate are usually interpreted as biological heterogeneity among patients due to, e.g., genetic variability. However, these variations might be a result of non-exponential, e.g. the Gompertzian, tumour growth kinetics. The aim was to study if the natural tumour growth deceleration, i.e. non-exponential growth, is a dominant factor in such variations.

**Materials and methods:**

The correlation between specific growth rate (SGR) and the logarithm of tumour volume, Ln(V), was calculated for tumours in patients with meningioma, hepatocellular carcinoma, pancreatic carcinoma, primary lung cancer, post-chemotherapy regrowth of non-small cell lung cancer (NSCLC), and in nude mice transplanted with human midgut carcinoid GOT1, a tumour group which is biologically more homogeneous than patient groups.

**Results:**

The correlation between SGR and Ln(V) was statistically significant for meningioma, post-chemotherapy regrowth of NSCLC, and the mouse model, but not for any other patient groups or subgroups, based on differentiation and clinical stage.

**Conclusion:**

This method can be used to evaluate the homogeneity of tumour growth kinetics among patients. Homogeneity of post-chemotherapy regrowth pattern of NSCLC suggests that, in contrast to untreated tumours, the remaining resistant cells or stem cells (if exist) might have similar biological characteristics among these patients.

## Introduction

Mathematical modelling of tumour growth can provide not only key insights into tumour biology but also tools for, e.g., optimization of screening programs, cancer patient prognosis [1], scheduling of chemotherapy [2], and assessment of tumour spread [3,4]. For example, Norton et al. showed that, considering the mathematical growth model of breast cancer tumours, patients must be treated with condensed-dose chemotherapy, and a clinical trial showed the significant benefit for the patients treated with the new method compared with the patients treated with the standard treatment [5,6].

However, one of the main limitations for using tumour growth rate in patient studies is that therapy is usually started soon after diagnosis and the natural growth of tumours can be followed only for a limited period of time, during which the growth of tumours is usually well described by the exponential model. Tumour volumes are usually estimated by delineating tumours in computed tomography (CT) or magnetic resonance imaging (MRI) slice, multiplying the measured area with slice thickness, and adding all volumes together. There are, therefore, uncertainties involved in estimated volumes in form of intra- and inter-investigator variations of estimated volumes. However, growth rate of exponentially growing tumours can be quantified with tumour volume doubling time (DT), given in days or months. However, the specific growth rate (SGR) of tumours, given in, e.g. percent per day, is mathematically more accurate and biologically more relevant than DT for quantification of tumour growth rate [7,8]. If the tumour volume is measured at times t_0_ and t, the following equation can be used for calculation of SGR [7]:

(A) $$\text{SGR}=\frac{\ln\left(V/V_{0}\right)}{t‒t_{0}},$$

where V_0_ and V are the volume of tumour at t = t_0_ and t, respectively.

According to the exponential model, the growth rate, i.e. SGR, is constant and independent of tumour age or volume. However, studies have shown that tumour growth rate may decelerate as tumour grows [9-11]. Growth deceleration has been observed in animal models [12], for solid tumours in clinical studies [13,14], and in leukaemia [9]. Growth deceleration is attributed to several factors, including prolonged cell cycle time, reduced growth fraction, decreased availability of oxygen [15], decreased cell proliferation rate with increased cell loss rate [16], tumour-related systemic factors [17], and allometric growth control [18]. Regardless of the mechanism of growth deceleration, a number of non-exponential growth models are available in the literature [19], among which the Gompertzian model is widely used. According to the Gompertzian growth model, the variation of tumour volume by time is as follows:

(B) $$V=V_{0}e^{\frac{\text{SG}R_{0}}{\lambda }\left(1‒e^{‒\lambda \left(t‒t_{0}\right)}\right)},$$

where SGR_0_ is the initial growth rate of tumour at t = t_0_ and λ is the growth deceleration constant.

Fitting the Gompertzian model to the natural growth of tumours needs at least three tumour volume values measured on occasions spread over a relatively long period of time, which is rarely obtainable in clinical observations. We have previously developed a method for estimation of growth deceleration constant, λ, from the linear regression of SGR with the logarithm of tumour volume [20]:

(C) $$\text{SGR}=\text{SG}R_{0}‒\lambda \ln\left(V/V_{0}\right)$$

The above equation enabled us estimating the growth model, including Gompertzian growth deceleration constant in Equation B (λ) and formation times of metastases in individual patients.

Tumour response to a specific treatment varies largely among patients. Beside other biological factors, tumour growth kinetics is important for how a tumour responds to therapy [21,22]. Tumour volume at base line and its growth rate have both been shown to be correlated with tumour response to therapy. Different factors are responsible for the varying tumour growth rates among patients, e.g., genetic factors, microenvironment in host tissue and growth deceleration as tumours grow. Furthermore, measurement uncertainties may also influence the reported variation in growth rate.

According to Equation C, a significant correlation between SGR and ln(V) in a group of tumours indicates that tumours probably follow the same growth curve and the difference in their growth rate is a result of difference in their volume. In other words, such a correlation is a measure of similarity of growth pattern among tumours, e.g., in a group of patients. Then, a unique curve can describe the growth of all tumours in the group, e.g., the curve that we derived for growth of liver metastases from a primary ileum carcinoid in a previous article [20].

The aim of this study was to assess the homogeneity of the growth kinetics of tumours of the same type in a population of patients. Data from different groups of patients, including meningioma, hepatocellular carcinoma, pancreatic carcinoma, and primary lung cancer, were analysed. The model was also applied to data from a mouse model bearing transplanted human midgut carcinoid GOT1 tumours, a tumour group which is biologically more homogeneous than patient groups.

## Materials and methods

### Calculation method

SGR was calculated for each pair of tumour volume measurements using Equation A. Correlation between SGR and ln(V) was calculated between all SGR values and their corresponding tumour volume, i.e., the geometric mean of the two volumes used for calculation of SGR.

### Clinical data

Data from clinical studies were retrieved from the literature based on the availability of tumour volume estimates and corresponding measurement time intervals. Tumour volumes were estimated by delineating tumours in computed tomography (CT) and/or magnetic resonance imaging (MRI) slices and multiplying the measured area with slice thickness. Correlation between SGR and the logarithm of the volume of tumours was calculated for the following types of tumours.

### Lung cancer

Data on the growth of non-small cell lung cancer (NSCLC) tumours in 18 patients were used [23]. Tumour growth was measured between the end of induction chemotherapy and the start of radiation therapy. The study showed that the regrowth of tumours after induction therapy (mean DT = 46 days, median DT = 29 days) was much faster than the untreated tumour growth rate found in the literature (mean DT range: 102–452 days). Of the potentially curable patients 41% became incurable in the waiting period between chemo- and radio-therapy. Tumour DT was shorter for smaller tumours compared to large tumour. Considering the fast regrowth of NSCLC tumours after induction chemotherapy, they recommended diminishing the time interval between chemo- and radiotherapy to as short as possible [23].

Another set of data was found for the growth rate and characteristics of small peripheral lung tumours as they appear on CT images. The tumour types included were rapidly growing (DT < 150 days) small cell lung cancer, adenocarcinoma, and squamous cell carcinoma tumours [24].

### Pancreatic carcinoma

Data for untreated pancreatic carcinoma in nine patients who underwent serial examinations by helical computed tomography was used for calculations [25]. The mean DT of the nine primary lesions was 159 days (median 144 days). Survival time was significantly correlated with DT.

### Hepatocellular carcinoma

The growth of hepatocellular carcinoma (HCC) was studied in 11 untreated patients where serial CT or MRI images were available (16 tumours in total) [26]. Calculated DT value range was 17.5-541 days and the mean DT was 127 days. DT was related to baseline volume as DT = 114 × (baseline volume)^0.14^. This study showed that smaller tumours had shorter DT values, i.e. grew faster, than larger tumours and, therefore, may require shorter follow-up time to observe progression [26].

In an analyses of data from 34 HCC patients, the growth rate of most tumours could be estimated properly only using histological parameters, e.g. Ki-67index, apoptotic index, and histologic grade, available at a single time point (DT range was 17–274 days) [27]. We determined SGR and tumour volumes based on data in Table 1 in that article [27].

**Table 1:** Correlation between the specific growth rate, SGR, and the logarithm of tumour volume in groups of patients diagnosed with the same type of tumour

**Tumour type**	**Reference**	**n**	**R**^**2**^	**p (1-tailed)**	**Median time interval (days)**	**Median time interval/DTe**	**Average SGR (%/d)**	**Relative uncertainty of SGR (%)**	**DTe (days)**	**Relative uncertainty of volume estimation (%)**
Meningioma	Nakamura et al. [29]	41	0.2424	**0.0005**	1230	0.71	0.04	105	1733	37
Hepatocellular carcinoma	Nakajima et al. [27]	34	0.038	NS (0.13)	128	2.4	1.30	73	53	86
	Saito et al. [28]	21	0.0623	NS (0.14)	146	1.05	0.50	47	139	24
	Taouli et al. [26]	16	0.0014	NS (0.89)	NA	NA	0.90	115	77	NA
Pancreatic carcinoma	Furukawa et al. [25]	9	0.0248	NS (0.34)	295	2.13	0.50	58	139	60
Primary Lung Cancer	Wang et al. [24]	12	0.1619	NS (0.19)	365	4.21	0.80	36	87	74
	El Sharouni et al. [23]	18	0.2713	**0.026**	48	1.94	2.80	73	25	69
	**REGROWTH**									

n: number of tumours.

R: correlation coefficient. Median time interval is calculated from measurement time intervals between the first and the second tumour volume measurements. NA: not available. NS: not statistically significant (p-values in parentheses). DT_e_: equivalent doubling time. DT_e_ and uncertainty calculations were done according to previously published methods [7]. Regrowth in the last row means that the calculation has been done for regrowth of tumours after induction chemotherapy.

Another data set on HCC included tumours less than 3 cm in diameter at first observation in 21 patients [28]. The natural progression of each lesion (DT) was observed by ultrasonography [28]. They concluded that data from cell kinetic parameters and histological grade are useful for estimating the natural growth rate of HCC. We determined SGR and tumour volumes based on data in Table 1 in that article [28].

### Meningioma

The natural history of incidental meningiomas was studied in asymptomatic patients [29]. Average tumour volume was 9 cm^3^ and DT ranged from 1.27 to 143 years (mean, 21.6 y). DT was shorter for younger patients, but was not correlated with tumour size. We determined SGR and tumour volumes based on data from that article [29]. The relatively large number of data included enabled us dividing tumours into two groups and comparing SGR between large and small tumours.

### Mouse model of human midgut carcinoid, GOT1

In order to test the validity of our method, the correlation between SGR and ln(V) was studied on tumours in the GOT1 nude mouse model (human midgut carcinoid tumours), which should biologically be more homogeneous compared with patients [30]. Data from 57 mice was gathered and the natural growth of tumours was followed for several weeks before any treatment.

### Statistical analyses

To test the significance of correlations, the t-values for Pearson correlation were calculated and then converted to p-values using one tailed T.DIST function in Microsoft Excel. P-value < 0.05 was assumed to be statistically significant. All patient data used in this study were retrieved retrospectively from already published articles. This type of information is exempt from ethical approval. The animal experiments were approved by the Ethical Committee for Animal Research at University of Gothenburg.

## Results

**Figure 1:**
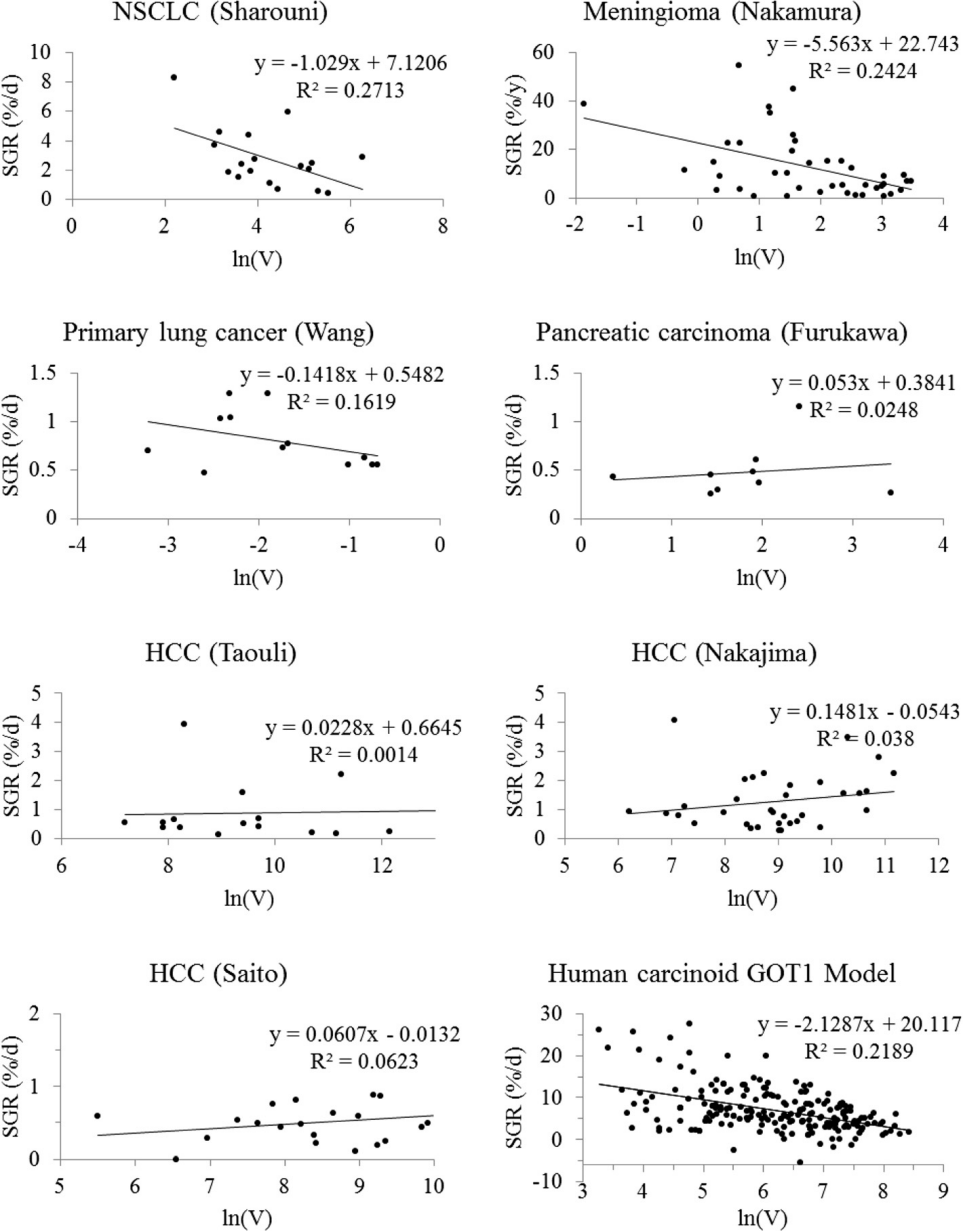
**Regression of specific growth rate, SGR, with the logarithm of tumour volume for post-chemotherapy regrowth of NSCLC (p < 0.03) and pre-treatment growth of meningioma tumours (p < 0.01), primary lung cancer (NS), pancreatic carcinoma (NS), hepatocellular carcinoma (NS), and human carcinoid GOT1 tumours in the mouse model (p < 1E-11).** NS: Not statistically significant.

**Figure 2:**
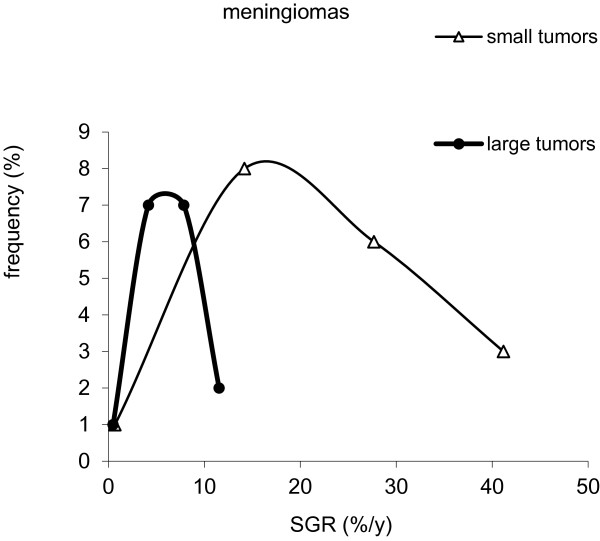
**Frequency distribution of specific growth rate, SGR, in two groups of small (n = 20) and large (n = 21) meningioma tumours.** Mean SGR was 20%/y and 6%/y for small and large tumours, respectively (p < 0.001).

The correlation between SGR and the logarithm of the volume of different types of tumours are shown in Figure 1 and Tables 1, 2 and 3. The correlation was statistically significant for meningiomas [29] and regrowth of non-small cell lung cancer tumours after induction chemotherapy [23]. The correlation was not statistically significant for the other patient groups and subgroups of tumours. The difference between the growth rate of the large and small tumours in meningioma group was statistically significant (p < 0.001), with higher SGR for smaller tumours (Figure 2). Mean SGR was 20%/y and 6%/y for small tumours (n = 20) and large tumours (n = 21), respectively.

**Table 2:** Correlation between the specific growth rate, SGR, and the logarithm of tumour volume in hepatocellular carcinoma patients

**Tumour type**	**Group**	**n**	**R**^**2**^	**p-value**
Hepatocellular carcinoma,				
Nakajima et al. [27]	WD	19	0.001	0.5
	MD	9	0.063	0.3
	PD	6	0.441	0.1
	CS I	17	0.040	0.2
	CS II	15	0.009	0.4
	WD & CS I	8	0.030	0.3
	WD & CS II	10	0.001	0.5

Tumours were grouped according to differentiation and clinical stage. None of the correlations were significant. WD: Well differentiated. MD: Moderately differentiated. PD: poorly differentiated. CS: Clinical stage. n: number of tumours. R: correlation coefficient.

**Table 3:** Correlation between the specific growth rate, SGR, and the logarithm of tumour volume in hepatocellular carcinoma patients

**Tumour type**	**Group**	**n**	**R**^**2**^	**p-value**
Hepatocellular carcinoma,				
Saito et al. [28]	WD	15	0.088	0.1
	MD	6	0.007	0.4

Tumours were grouped according to differentiation level. None of the correlations were statistically significant. WD: Well differentiated. MD: Moderately differentiated. n: number of tumours. R: correlation coefficient.

The correlation between SGR of tumour and the logarithm of its volume was statistically significant for data from nude mice bearing GOT1 tumours (p < 1E-11) (Figure 1).

## Discussion

Accurate quantification and analyses of the tumour growth rates is essential for understanding the biological variance of human cancers [31]. Differences in the observed growth rates of tumours of the same type in a population of patients can be due to: (a) measurement uncertainties, (b) growth deceleration with increasing tumour volume, or (c) other biological differences between tumours, e.g., their location and microenvironment. With regard to measurement uncertainties, we previously showed that SGR is the tumour growth rate measure that is least influenced by measurement errors, compared to other growth rate measures, e.g., tumour volume doubling time (DT) [7]. We also showed that SGR is least affected by variances due to biological factors [8]. In the present study, focused on the growth deceleration in tumours, we used the relation between SGR and the logarithm of tumour volume to assess the contribution of growth rate decline in the observed variances in tumour growth rate found in the selected clinical studies. It should be noted that the limited amount of data on natural tumour growth available makes this type of studies difficult.

Expected highly significant negative correlation between SGR and the logarithm of tumour volume in the mouse model (which should be biologically more homogeneous compared to tumours in groups of patients) showed that the presented method is a useful tool to assess if growth deceleration is an important factor influencing difference in tumour growth rates observed among patients.

A significant correlation between SGR and the logarithm of tumour volume in a group of tumours also indicates that the smaller tumours represent the growth of larger tumours when they were of small size and *vice versa*. This provides the possibility for further development of mathematical models for elaborating this correlation in more accurate efficacy assessment of new drugs or combination of treatments. However, lack of correlation between SGR and the logarithm of tumour volume indicates that biological factors other than growth deceleration are more important for explaining the differences in the tumour growth rate observed in a population of patients with the same tumour type. These tumours may grow exponentially with different growth rates, or according to the Gompertzian model, and the model constants, SGR_0_ and λ, are heterogeneously distributed among tumours [20].

In this study, the correlation between SGR and the logarithm of tumour volume was statistically significant for the growth of tumours in meningioma patients. Further analysis by dividing the material into small and large tumours also supported this result. A similar growth model observed for a group of patients with one tumour type corroborates that the response rate in this group might be a suitable measure to assess the efficacy of novel treatments. The correlation between tumour growth rate and the logarithm of its volume was, however, not statistically significant for the studied patient cohorts with hepatocellular carcinoma, pancreatic carcinoma, and primary lung cancer. This result was expected for the primary lung cancer cohort, since that included different types of lung cancer. A lack of correlation indicates that the growth of these types of tumours varies between patients with similar tumour type and, therefore, growth rate should be taken into account as an independent variable in efficacy assessment of treatment of patients with these types of tumours both in clinical trials and for individualized therapy planning.

However, in the present study we were in general not able to include factors such as histologic grade and differentiation in our analyses, due to the limited number of tumours/patients or lack of information for other types of tumours. The only exception was for hepatocellular carcinoma, where subgroups could be categorized according to their differentiation level and clinical stage. However the correlation between tumour growth rate and the logarithm of its volume was not statistically significant for any of the two patient cohorts studied. It would otherwise be possible that stratification of the tumours according to, e.g., histopathological information would have given better correlation within subgroups of each tumour type. The result of the animal study indicates such a situation.

Nevertheless, the significant correlation between tumour growth rate and the logarithm of its volume for regrowth of primary lung cancer tumours after chemotherapy was interesting, because this can be interpreted as homogeneity of these tumours among patients. Post-chemotherapy tumours consisting of resistant cancer cells or cancer stem cell clones (if exist) of non-small cell lung cancer might be more homogeneous in terms of cellular and histological characteristics and, therefore, respond more similarly to therapy. Such information is valuable for further treatments in, e.g., neoadjuvant therapy.

The main difference between meningioma and other types of tumours in the present study is that meningiomas are benign. It is possible that the reason for meningioma being the only tumour type that gave significant correlation between tumour size and tumour growth rate in the patient cohorts is that it is benign, with low proliferation rate and histologically more homogeneous than malignant tumours.

In the original study on meningioma, no correlation between tumour DT and its volume was found [29]. On the other hand, we found strong correlation between SGR and the logarithm of tumour volume, a correlation that, by definition, is the accurate measure of growth rate deceleration as tumour grows. The significant difference between growth rate of small and large tumours also supported our result. This is in line with our previous study where we showed that using DT for quantification of tumour growth rate can result in wrong conclusions [8]. This emphasizes again the need for developing new accurate mathematical tools to analyse clinical data.

In conclusion, the presented method, i.e. estimation of the Gompertzian growth deceleration constant using limited clinical data, can be used to evaluate the homogeneity of tumour growth pattern among patients. Tumour growth kinetics was largely heterogeneous among patients with the same type of tumour, except for the meningioma group. This implies that the tumour growth kinetics in each patient should be considered in efficacy assessment of new drugs and for optimization of treatment in individual patients using, e.g., the tumour response model in ref [21,22]. Furthermore, homogeneity of post-chemotherapy regrowth pattern of non-small cell lung cancer tumours suggests that, in contrast to pre-treatment tumours, the remaining resistant cancer cells or cancer stem cells (if exist) might have similar biological characteristics among these patients, a factor that should be valuable to consider in, e.g., neoadjuvant therapy.

## Competing interests

The authors declare that they have no competing interests.

## Authors’ contributions

EM and EFA initiated the study. EM developed the model and analyzed the data. EM and EFA drafted the manuscript. Both authors read and approved the final manuscript.
